# Retraction

**DOI:** 10.1111/cas.15809

**Published:** 2023-05-22

## Abstract

Retraction: Yang, Y, Zheng, J, Wang, M, et al. NQO1 promotes an aggressive phenotype in hepatocellular carcinoma via amplifying ERK‐NRF2 signaling. Cancer Sci. 2021; 112: 641–654. https://doi.org/10.1111/cas.14744.

The above article, published online on 22 November 2020 in Wiley Online Library (wileyonlinelibrary.com), has been retracted by agreement between the authors, the journal's Editor‐in‐Chief, Masanori Hatakeyama, the Japanese Cancer Association and John Wiley and Sons Australia, Ltd. The retraction has been agreed following concerns raised by a third party about figures within the article. During the journal's investigation into the concerns raised, the authors were not able to provide comprehensive original data for the figures in concern. Accordingly, the editorial team considers the conclusions of this manuscript insufficiently supported.

Retraction: Yang, Y, Zheng, J, Wang, M, et al. NQO1 promotes an aggressive phenotype in hepatocellular carcinoma via amplifying ERK‐NRF2 signaling. Cancer Sci. 2021; 112: 641–654. https://doi.org/10.1111/cas.14744.

The above article, published online on 22 November 2020 in Wiley Online Library (wileyonlinelibrary.com), has been retracted by agreement between the authors, the journal's Editor‐in‐Chief, Masanori Hatakeyama, the Japanese Cancer Association and John Wiley and Sons Australia, Ltd. The retraction has been agreed following concerns raised by a third party about figures within the article. During the journal's investigation into the concerns raised, the authors were not able to provide comprehensive original data for the figures in concern. Accordingly, the editorial team considers the conclusions of this manuscript insufficiently supported.

